# On weighted *k*-mer dictionaries

**DOI:** 10.1186/s13015-023-00226-2

**Published:** 2023-06-17

**Authors:** Giulio Ermanno Pibiri

**Affiliations:** 1 Department of Environmental Sciences, Informatics and Statistics (DAIS), Ca’ Foscari University of Venice, Venice, Italy; 2 ISTI-CNR, Pisa, Italy

**Keywords:** *k*-mers, Compression, Hashing, Graphs, Path cover

## Abstract

We consider the problem of representing a set of $k$-mers and their abundance counts, or weights, in compressed space so that assessing membership and retrieving the weight of a $k$-mer is efficient. The representation is called a *weighted dictionary* of $k$-mers and finds application in numerous tasks in Bioinformatics that usually count $k$-mers as a pre-processing step. In fact, $k$-mer counting tools produce very large outputs that may result in a severe bottleneck for subsequent processing. In this work we extend the recently introduced SSHash dictionary (Pibiri in Bioinformatics 38:185–194, 2022) to also store compactly the weights of the $k$-mers. From a technical perspective, we exploit the order of the $k$-mers represented in SSHash to encode *runs* of weights, hence allowing much better compression than the empirical entropy of the weights. We study the problem of reducing the number of runs in the weights to improve compression even further and give an optimal algorithm for this problem. Lastly, we corroborate our findings with experiments on real-world datasets and comparison with competitive alternatives. Up to date, SSHash is the only $k$-mer dictionary that is exact, weighted, associative, fast, and small.

## Introduction

Recent advancements in the so-called Next Generation Sequencing (NGS) technology made possible the availability of very large collections of DNA. However, before being able to actually analyze the data at this scale, efficient methods are required to index and search such collections. One popular strategy to address this challenge is to consider short sub-strings of fixed length *k*, known as $k$-mers. Software tools based on $k$-mers are predominant in Bioinformatics and they have found applications in genome assembly [1, 2], variant calling [3, 4], pan-genome analysis [5, 6], meta-genomics [7], sequence comparison [8–10], just to name a few ones.

For several such applications it is important to quantify how many times a given $k$-mer is present in a DNA database. In fact, many efficient $k$-mer counting tools have been developed for this task [11–15]. The output of these tools is a table where each distinct $k$-mer in the database is associated to its abundance count, or *weight*. The weights are either exact or approximate (in this work, we focus on exact weights). These genomic tables are usually very large and take several GBs—in the range of 40–80 bits/$k$-mer or more according to recent experiments [13, 16, 17]. Therefore, the tables should be compressed effectively while permitting efficient random access queries in order to be useful for on-line processing tasks. This is precisely the goal of this work. We better formalize the problem as follows.

### **Problem 1**

(*The Weighted* $k$*-mer Dictionary Problem*) Let *S* be a long DNA string and $\mathcal {K}$ be the set of the *n* distinct pairs $\langle g, w(g) \rangle$, where *g* is a $k$-mer of *S* and *w*(*g*) is the *weight* of *g*, i.e., the number of times *g* occurs in *S*. We want a compressed representation of $\mathcal {K}$ that permits to efficiently check the *exact* membership of *g* to $\mathcal {K}$ and, if *g* actually belongs to $\mathcal {K}$, retrieve *w*(*g*).

In a previous investigation, we proposed a *sparse and skew hashing* scheme for $k$-mers (SSHash, henceforth) [18]—a compressed dictionary that relies on $k$-mer *minimizers* [8] and *minimal perfect hashing* [19, 20] to support fast membership (in both random and streaming query modality) in succinct space. These features make SSHash useful for several applications, including reference indexing and pseudo-alignment [21]. However, we did not consider the weights of the $k$-mers. In this work, therefore, we enrich the SSHash data structure with the weight information to solve Problem 1. The main practical result is that, by exploiting the *order* of the $k$-mers represented in SSHash, the compressed exact weights take only a small extra space on top of the space of SSHash. This extra space is proportional to the number of *runs* (maximal sub-sequences formed by all equal symbols) in the weights and not proportional to the number of distinct $k$-mers. As a consequence, the weights are represented in a much smaller space than the empirical entropy lower bound.

We study the problem of reducing the number of runs in the weights and model it as a *graph covering* problem, for which we give an optimal, linear-time, algorithm. The optimization algorithm effectively reduces the number of runs to the minimum, hence improving space even further.

When empirically compared to other weighted dictionaries that can be either somewhat smaller but much slower or much larger, SSHash embodies a robust trade-off between index space and query efficiency.

## Related work

A solution to the weighted $k$-mer dictionary problem can be obtained using the popular FM-index [22]. The FM-index represents the original DNA string taking the Burrows-Wheeler transform (BWT) [23] of the string. Reporting the weight of a $k$-mer is solved using the *count* operation of the FM-index which involves *O*(*k*) rank queries over the BWT.

Another solution using the BWT is the so-called BOSS data structure [24] that is a succinct representation of the *de Bruijn* graph of the input—a graph where the nodes are the $k$-mers and the edges model the overlaps between the $k$-mers. The BOSS data structure has been recently enriched with the weights of the $k$-mers [16], by delta-encoding the weights on a spanning branching of the graph. Since consecutive $k$-mers often have equal (or very similar) weights, good space effectiveness is achieved by this technique.

Other solutions, instead, rely on hashing for faster query evaluation compared to BWT-based indexes. For example, both deBGR [17] and Squeakr [13] use a *counting quotient filter* [25] to store the $k$-mers and the weights. They can either return approximate weights, i.e., wrong answers with a prescribed (low) probability, for better space usage of exact weights at the price of more index space. In any case, the memory consumption of these solutions is not competitive with that of BWT-based ones as they do not employ sophisticated compression techniques and were designed for other purposes, e.g., dynamic updates.

A closely related problem is that of realizing *maps* from $k$-mers to weights, i.e., data structures that do *not* explicitly represent the $k$-mers and so return arbitrary answers for out-of-set keys. In the context of this work, we distinguish between such approaches, *maps*, and dictionaries that instead represent *both* the $k$-mers and the weights. Besides minimal perfect hashing [19, 20], some efficient maps have been proposed and tailored specifically for genomic counts, such as based on *set-min sketches* [26] and *compressed static functions* (CSFs) [27]. These proposals leverage on the repetitiveness of the weights (low-entropy distributions) to obtain very compact space.

Lastly in this section, we report that other works [28, 29] considered the multi-document version of the problem studied here, that is, how to retrieve a vector of weights for a query $k$-mer, where each component of the vector represents the weight of the $k$-mer in a distinct document. Also such count vectors are usually very “regular” (or can be made so by introducing some approximation) [28] and present runs of equal symbols that can be compressed effectively with *run-length encoding* (RLE).

## Representing runs of weights

In this section we describe the compression scheme for the weights that we use in SSHash. Recall that we indicate with $\mathcal {K}$ the set of *n* distinct $\langle$ $k$-mer, weight$\rangle$ $=$ $\langle g, w(g) \rangle$ pairs, that we want to store in a dictionary. With a little abuse of notation, we write “$g \in \mathcal {K}$” for a $k$-mer *g* to mean that there is a pair of $\mathcal {K}$ whose $k$-mer is *g*. We first highlight the main properties of SSHash that we are going to exploit in the following to obtain good space effectiveness for the weights (for all the other details concerning the SSHash index, we point the interested reader to our previous work [18]).

From a high-level perspective, SSHash implements the function $h: \Sigma ^k \rightarrow \{0,1,\ldots ,n\}$, where $n=|\mathcal {K}|$ and $\Sigma ^k$ is the whole set of *k*-length strings over the DNA alphabet $\Sigma =\mathsf {\{A,C,G,T\}}$. In particular, *h*(*g*) is a unique value $1 \le i \le n$ if $g \in \mathcal {K}$; or $h(g)=0$ otherwise, i.e., if $g \notin \mathcal {K}$. In other words, SSHash serves the same purpose of a minimal and perfect hash function (MPHF) [20] for $\mathcal {K}$ but, unlike a traditional MPHF, SSHash *rejects* alien $k$-mers. This is possible because the $k$-mers of $\mathcal {K}$ are actually represented in SSHash whereas the space of a traditional MPHF does not depend on the input keys.

The value $i=h(g)$ for the $k$-mer $g \in \mathcal {K}$ is the handle of *g*, or its “hash” code. The hash codes can be used to associate some satellite information to the $k$-mers such as, for example, the weights themselves using an array *W*[1..*n*] where $W[h(g)]=w(g)$.

SSHash takes advantage of the fact that the input set $\mathcal {K}$ can be processed into a so-called *spectrum-preserving string set* (or SPSS) $\mathcal {S}$—a collection of strings $\mathcal {S}=\{{S}_1,\ldots ,{S}_m\}$ where each $k$-mer of $\mathcal {K}$ appears exactly once. We omit the details here on how the collection $\mathcal {S}$ can be built; we only report that there are efficient algorithms for this purpose that also try to minimize the total number of symbols in $\mathcal {S}$, i.e., the quantity $\sum _{i=1}^m |{S}_i|$. One such algorithm is the UST algorithm [30] that we also use to prepare the input for SSHash. The key property of SSHash in which we are interested is that—after $\mathcal {K}$ is processed into the SPSS $\mathcal {S}$—the function *h* *preserves the relative order* of the $k$-mers, that is: if $g_1[1..k]$ and $g_2[1..k]$ are two $k$-mers with $g_1[2..k]=g_2[1..k-1]$ (i.e., $g_2$ comes immediately after $g_1$ in a string), then $h(g_2)=h(g_1)+1$. Therefore, consecutive $k$-mers, i.e., those sharing an overlap of $k-1$ symbols, are also given consecutive hash codes.

Therefore, once an order ${S}_1,\ldots ,{S}_m$ for the strings of $\mathcal {S}$ is fixed, then also an order $i=1,\ldots ,n$ for the $k$-mers $g_i$ is uniquely determined. Let *W*[1..*n*] be the sequence of weights in this order. Then, we have: $h(g_i)=i \text { and } W[i] = w(g_i), \text { for } i=1,\ldots ,n.$

This order-preserving behavior of *h* induces a property on the sequence of weights *W*[1..*n*] that significantly aids compression: *W* contains *runs*, i.e., maximal sub-sequences of *equal weights*. This is so because consecutive $k$-mers are very likely to have the same weight due to the high specificity of the strings. This a known fact, also observed in prior work [16, 27, 28]. Here, we are exploiting the order of the $k$-mers given by SSHash to preserve the natural order of the weights in *W*. Note that this cannot be achieved by approximate schemes that do not represent the $k$-mers themselves, like a generic MPHF or a CSF. Even if the $k$-mers were available, those schemes are unable to assign consecutive hashes to consecutive $k$-mers, actually shuffling the weights at random and, thus, making *W* very difficult to compress.

**Fig. 1:**
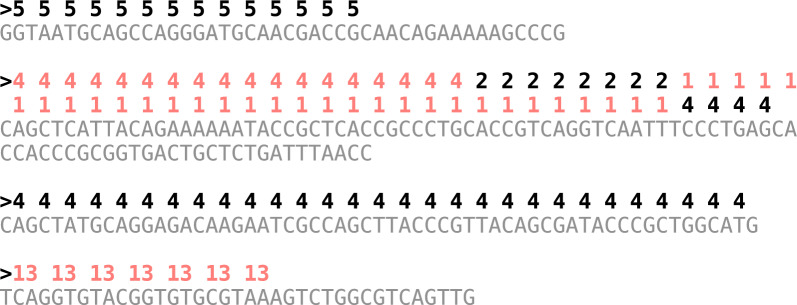
An example collection $\mathcal {S}$ of 4 weighted sequences (for $k=31$) drawn from the genome of *E. coli* (Sakai strain). With alternating colors we render the change of weight in the runs. There are 111 $k$-mers in the example but just 6 runs in the weights: $RLW = \langle 5,14 \rangle \langle 4,18 \rangle \langle 2,8 \rangle \langle 1,31 \rangle \langle 4,33 \rangle \langle 13,7 \rangle .$ Note that a run can cross the boundary between two (or more) sequences, as it happens for the run $\langle 4,18 \rangle$ which covers completely the third but also the part of the second sequence

It is standard to represent a sequence *W* featuring *r* runs of equal symbols using *run-length encoding* (RLE), i.e., *W* is modeled as a sequence of run-length pairs $RLW = \langle w_1,\ell _1 \rangle \langle w_2,\ell _2 \rangle \cdots \langle w_r,\ell _r \rangle$ where $w_i$ and $\ell _i$ are, respectively, the value of the run and the length of the *i*-th run in *W*. Figure 1 shows an example of *RLW* for a collection $\mathcal {S}$ with 4 weighted strings.

### Encoding RLW

 Let $\mathcal {D}$ be the set of all distinct $w_i$ in *RLW*. Clearly, $r \ge |\mathcal {D}|$ as we must have at least one run per distinct weight. We store $\mathcal {D}$ using $|\mathcal {D}|\lceil \log _2max \rceil$ bits where $max \ge 1$ is the largest $w_i$. We use $\mathcal {D}$ to uniquely represent each $w_i$ in *RLW* with $\lceil \log _2|\mathcal {D}|\rceil$ bits. Since runs are maximal sub-sequences in *W* by definition, then $w_i \ne w_{i+1}$ for every $i=1,\ldots ,r-1$ (adjacent weights must be different). Then we take the prefix-sums of the sequence $0,\ell _1,\ldots ,\ell _{r-1}$ into an array *L*[1..*r*] and encode it with Elias-Fano [31, 32].^1^ By construction we have that $\sum _{i=1}^r \ell _i = n$ since the runs must cover the whole set of $k$-mers. So the largest element in *L* is actually $n-\ell _r$ and we spend at most $r\lceil \log _2(n/r)\rceil + 2r + o(r)$ bits for *L*. Summing up, we spend at most

$$\begin{aligned} r \cdot \Big ( \lceil \log _2|\mathcal {D}|\rceil + \Big \lceil \log _2\Big (\frac{n}{r}\Big )\Big \rceil + 2 + o(1)\Big ) + |\mathcal {D}|\lceil \log _2max \rceil \text { bits} \end{aligned}$$

for representing *RLW* on top of the space of SSHash. In conclusion, the weights are represented in space proportional to the number of runs in *W* (i.e., $r=|RLW|$) and *not* proportional to the number of $k$-mers, which is *n*. As a consequence, this space is likely to be considerably less than the empirical entropy $H_0(W)$ as we are going to see with the experiments in “Experiments” section.

To retrieve the weight *w*(*g*) from $i=h(g)$, all that is required is to identify the run containing *i*. This operation is done in $O(\log (n/r))$ time with a predecessor query over *L* given that we represent *L* with Elias-Fano. If the identified run is the *j*-th run in *W*, then $w_j$ is retrieved in *O*(1) from $\mathcal {D}$.

## The problem of reducing the number of runs

In “Representing runs of weights” section we presented an encoding scheme for the $k$-mer weights whose space is proportional to the *number of runs* in the sequence of weights *W*. Therefore, in this section we consider the problem of reducing the number of runs in the weights to optimize the space of the encoding.

### Rules of the game

 We assume that the strings in $\mathcal {S}$ are *atomic* entities: it is not allowed to partition them into sub-strings (e.g., in correspondance of the runs of weights in the strings). In fact, since the strings are obtained by the UST algorithm [30] with the purpose of *minimizing* the number of nucleotides as we explained in “Representing runs of weights” section, breaking them will lead to an increased space usage for the $k$-mers, actually dwarfing any space-saving effort spent for the weights. With this constraint specified, there are only *two* degrees of freedom that can be exploited to obtain better compression for *W*: (1) the *order* of the strings, and (2) the *orientation* of the strings. Altering $\mathcal {S}$ using these two degrees of freedom does not affect the correctness nor the (relative) order-preserving property of the function $h: \Sigma ^k \rightarrow \{0,1,\ldots ,n\}$ implemented by SSHash. In fact, as evident from our description in “Representing runs of weights” section, the output of *h* will still be $\{1,\ldots ,n\}$ as the $k$-mers themselves do *not* change (even when taking reverse-complements into account as they are considered to be identical). What changes is just the absolute order of the $k$-mers as a consequence of permuting the order of the strings $\{{S}_1,\ldots ,{S}_m\}$ in $\mathcal {S}$.

**Fig. 2:**
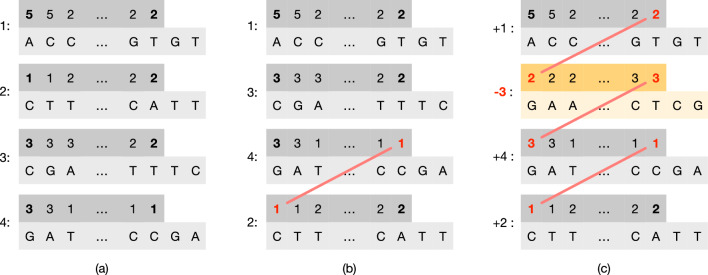
In **a**, an example input collection $\mathcal {S}$ of $m=|\mathcal {S}|=4$ weighted strings (for $k=3$), where the end-point weights are highlighted in bold font. In **b**, the order of the strings is changed according to the permutation $\pi =[1,4,2,3]$ and, as a result, the number of runs is reduced by 1 (the last run in string 4 is glued with the first run of string 2). Lastly, in **c**, it is shown that changing the orientation of string 3 (taking the reverse complement of the string and reversing the order of the $k$-mer weights) makes it possible to glue other two runs. Given that reducing the number of runs by $m-1$ is the best achievable reduction, the number of runs in **c** is therefore the minimum for the original collection in **a**


Therefore, our goal is to permute the order of the strings in $$\mathcal {S}$$ and possibly change their orientations to reduce the number of runs in *W*. We now consider an illustrative example to motivate why both these two operations—those of changing the order and orientation of a string—are important to reduce the number of runs. Refer to Fig. 2a which shows an example collection of $$m=|\mathcal {S}|=4$$ weighted strings (for $$k=3)$$. Applying the permutation $$\pi =[1,4,2,3]$$ as shown in Fig. 2b reduces the number of runs by 1 because the run at the junction of string 4 and 2 can be glued. Lastly, applying the *signed* permutation $$\pi =[+1,+4,-2,+3]$$ as in Fig. 2c reduces the number of runs by 3, which is the best possible. Our objective is to compute such a signed permutation $$\pi $$ for an input collection of strings, in order to permute $$\mathcal {S}$$ as shown in Algorithm 1.

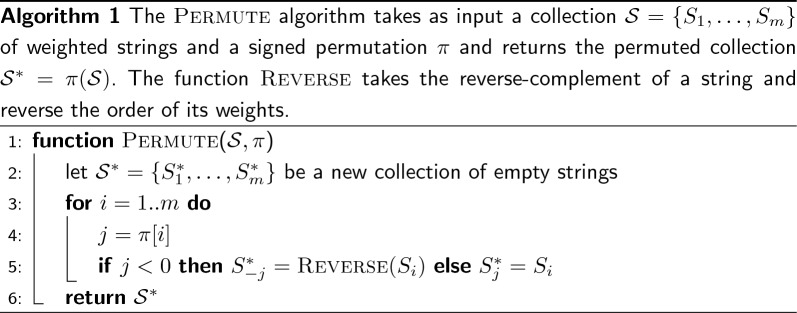

**Fig. 3:**
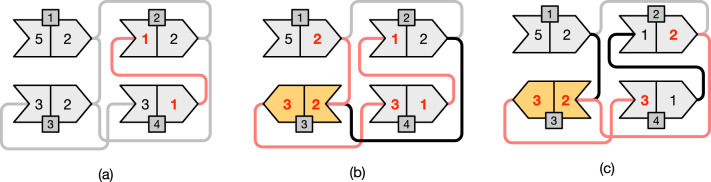
The same example of Fig. 2 but modeled using end-point weight graphs. Each node is represented using an arrow-like shape with two-matching sides. Only opposite sides having the same weight can be matched. The numbers inside the shapes represent the end-point weights; the extra darker square contains the node identifier. An arrow oriented from *left-to-right* models a node with *positive* sign; vice versa, an arrow oriented from *right-to-left* models a node with *negative* sign. Gray edges represent edges that *cannot* be traversed without changing the orientation of one of the two connected nodes. Black edges represent edges that can be traversed. Lastly, we highlight in red the edges that belong to paths in a graph cover. The example in **a** corresponds to that of Fig. 2b where no node has changed orientation and, therefore, we have three paths in the cover: $(+4 \rightarrow +2)$, $(+3)$, and $(+1)$. Other two different covers are shown in **b** and **c**. In **b** the cover contains the single path $(+1 \rightarrow -3 \rightarrow +4 \rightarrow +2)$ and corresponds to the example of Fig. 2c where the node 3 was changed orientation from $+$ to—(shown in yellow color). In **c** the cover contains the two paths $(+2 \rightarrow -3 \rightarrow +4)$ and the singleton path $(+1)$

Figure 2 also suggests that the final result $\pi$ solely depends on the weight of the first and last $k$-mer of each sequence—which we call the *end-point* weights (or just end-points) of a sequence—and not on the other weights nor the nucleotide sequences. Therefore, it is useful to model an input collection $\mathcal {S}$ using a graph, defined as follows.

#### **Definition 1**

(*End-point weight graph*) Given a collection of weighted sequences $\mathcal {S}$, let *G* be a graph where:

- There is a node *u* for each sequence of $\mathcal {S}$ and *u* has two sides—a *front* and a *back* side—respectively labelled with the first and last weights of the sequence (end-point weights).
- There is an edge between any two nodes *u* and *v* that have a side with the same weight.

The graph *G* is called the end-point weight graph for $\mathcal {S}$ and indicated with $G(\mathcal {S})$.

In the following, we assume the collection $\mathcal {S}$ to be clear from the context and we refer to $G(\mathcal {S})$ simply as *G*. We indicate a node $u \in G$ using the identifier (*id*) of the corresponding sequence of $\mathcal {S}$. Also, we associate to *u* a $sign \in \{-1,+1\}$ (also called “orientation”), indicating whether the sequence should be reverse-complemented. In summary, a node $u \in G$ is the 4-tuple $(id ,front ,back ,sign )$.

#### **Definition 2**

(*Oriented path*) An *oriented* path of length $$\ell $$ in *G* is either a single node ($$\ell =1$$) or a sequence of nodes $$u_1 \rightarrow \cdots \rightarrow u_{\ell }$$ where each consecutive pair of nodes $$u_i \rightarrow u_{i+1}$$ is oriented in such a way that $$u_i.back = u_{i+1}.front $$, for any $$1 \le i < \ell $$, $$\ell \ge 2$$.

Since we will be interested only in oriented paths, we just refer to them as “paths”. For ease of notation, we will indicate a path in our examples as a sequence of signed numbers $$(i_1 \rightarrow \cdots \rightarrow i_{\ell })$$ where each number represents a node’s *id* and its sign represents the node’s *sign*. The first and the last node of a path *P* are indicated, respectively, with the $$P.front $$ and the $$P.back $$. The weights $$P.front .front $$ and $$P.back .back $$ are the two end-points of the path.

Given this graph model, it follows that the problem of finding a signed permutation $\pi$ for $\mathcal {S}$ is equivalent to that of computing a (disjoint-node) *path cover* *C* for *G*, i.e., a set of paths in *G* that visit *all* the nodes and where each node belongs to *exactly one* path. In fact note that, given a cover *C* for *G*, there is a linear-time reduction from *C* to $\pi$ as illustrated in Algorithm 2. Since the cover *C* is a disjoint-node path cover, the correctness of the algorithm is immediate as well as its complexity of $\Theta (m)$.
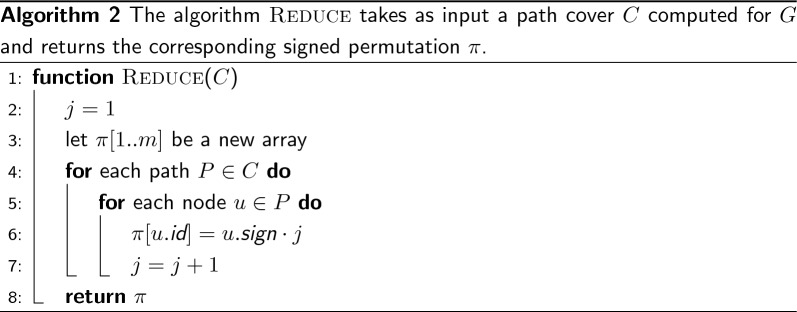


Figure 3 illustrates the same example of Fig. 2 but with end-point weight graphs. In Fig. 3b we would obtain a cover $C=\{(+1 \rightarrow -3 \rightarrow +4 \rightarrow +2)\}$ formed by a single path. In this case the permutation $\pi$, following Algorithm 2, would be $\pi [1]=+1$, $\pi [3]=-2$, $\pi [4]=+3$, and $\pi [2]=+4$. This is indeed the same permutation discussed in Fig. 2c. Another example: for the graph in Fig. 3c, the cover would be $C=\{(+2 \rightarrow -3 \rightarrow +4),(+1)\}$ and the permutation $\pi$ would be $\pi [2]=+1$, $\pi [3]=-2$, $\pi [4]=+3$, and $\pi [1]=+4$.

## Computing a minimum-size path cover

We showed that changing the order and orientation of the strings in $\mathcal {S}$ can reduce the number of runs in the weights. The question we now ask is: *by how much?* We are interested in computing an optimal signed permutation $\pi$ for $\mathcal {S}$, i.e., a permutation $\pi$ such that $\pi (\mathcal {S})$ has the minimum number of runs. Since we modeled the problem of computing $\pi$ as the problem of finding a path cover for *G*, we reason in terms of *G* and a path cover *C* for *G*.

Let $r_i$ be the number of runs in ${S}_i$ and let *R* be the total number of runs, i.e., $R=\sum _{i=1}^{m} r_i$. Let also |*C*| be the number of paths in the cover *C*. We observe that the final number of runs *r* in the permuted $\mathcal {S}$ will be equal to $R-m+|C|$. In fact, every path in *C* must begin (resp. end) with a node whose front side (resp. back side) cannot be glued with any other path’s side. Therefore, a new run begins with the first node of every path. Since we wish to minimize the quantity $R-m+|C|$, and considering that $R-m$ is constant for a given $\mathcal {S}$, it follows that the problem reduces to that of minimizing |*C*|, the number of paths in the cover. In other words, the problem of minimizing the number of runs *r* is equivalent to that of finding a minimum-cardinality path cover *C* for *G*.

We recall that the problem of computing a minimum-cardinality path cover for directed graphs is NP-hard. However, in this section we show that the problem can be solved optimally using linear time and space on end-point weight graphs. Specifically, we explain the steps of the min-cover algorithm (Algorithm  3) and prove its optimality.
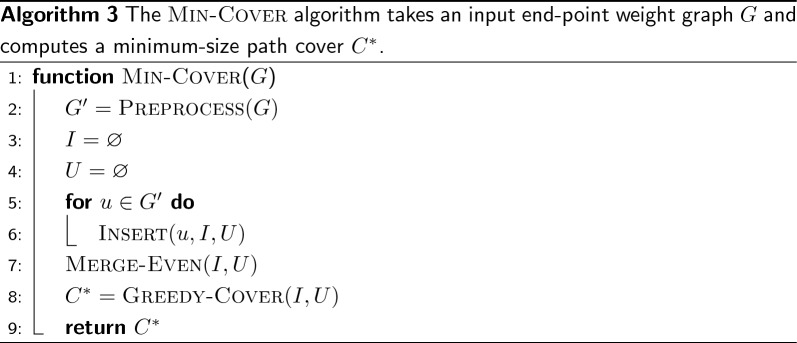


### Preliminaries and notation

Our focus is on the end-point weights of the nodes, thus throughout “Computing a minimum-size path cover” section we will denote a node $$(id ,front ,back ,sign )$$ just by its weights $$(front ,back )$$. Without loss of generality, we assume that each $$u \in G$$ is such that $$u.front \le u.back $$ since we can change the orientation of *u* if $$u.front > u.back $$ with a primitive change-orientation. In this way, two nodes (*x*, *y*) and (*y*, *x*) are considered as equal regarding their end-points.

Let $\mathcal {W}(G)$ be the set of the distinct end-point weights of *G* and $C^*(G)$ a minimum-size (i.e., optimal) path cover for *G*. Whenever clear from the context, we will avoid specifying *G* and refer to $\mathcal {W}(G)$ and $C^*(G)$ simply as $\mathcal {W}$ and $C^*$ respectively. Let |*G*| denote the number of nodes in *G*.

#### **Definition 3**

(*Incidence set and weight frequenc*y) The set $I_w$ of nodes where *w* appears as end-point is called the incidence set of *w*, for every $w \in \mathcal {W}$. We call the frequency of *w* the number of times *w* appears in the nodes of $I_w$ and indicate this quantity with $n(I_w)$.

**Fig. 4:**
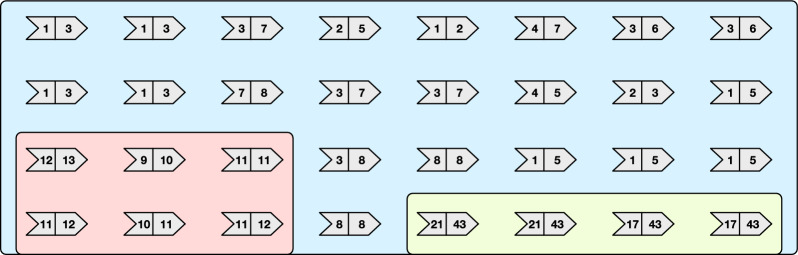
An end-point weighted graph *G* with $m=32$ nodes that is used as example throughout this section. The graph has 3 connected components, highlighted with different background colors, and $|\mathcal {W}|=16$ distinct weights

#### *Example 1*

Consider the graph *G* in Fig. 4. The graph has $m=32$ nodes, 3 connected components, and $|\mathcal {W}|=16$ distinct weights. For example, the incidence set for the weight 3 is

$$\begin{aligned} I_3 = \{(1,3),(1,3),(1,3),(1,3),(3,7),(3,7),(3,7),(3,6),(3,6),(2,3)\} \end{aligned}$$

and $n(I_3)=|I_3|=10$. Another example: $I_8=\{(3,8),(8,8),(8,8)\}$ and $n(I_8)=5$ but $|I_8|=3$.

### Graph simplification

In general, a path $u_1 \rightarrow \cdots \rightarrow u_{\ell }$ in *G* can be logically replaced by the single node of endpoints $(u_1.front ,u_{\ell }.back )$. When we do so, we say that *G* is “simplified” to a new graph where nodes $\{u_1,\ldots ,u_{\ell }\}$ are removed and the single node $(u_1.front ,u_{\ell }.back )$ is added. (When $\ell =2$ and nodes *u* and *v* are merged on weight *w*, we refer to this operation as $\textsc {Merge}({u,v,w})$ (see Algorithm 5). However, we remark that the simplification is only logical and not physical, i.e., the nodes removed from *G* are not truly discarded since each node of *G* must appear once in $C^*$. We therefore assume that the new node $(u_1.front ,u_{\ell }.back )$ keeps track of its inner structure and, when visited (e.g., in the for-loop in lines 5–7 of Algorithm 2), it actually visits each node in the path $u_1 \rightarrow \cdots \rightarrow u_{\ell }$.

#### **Lemma 1**

*Let* $E_{x,y}$ *be the subset of all equal nodes* (*x*, *y*) *of* *G*. *Let* $d=|E_{x,y}|$. *If* *d* *is even, then the* *d* *nodes can be oriented to form a maximal path of either end-points* (*x*, *x*) or (*y*, *y*). *If* *d* *is odd, then the path has end-points* (*x*, *y*).

#### *Proof*

We proceed by induction on *d*. Base case: if $d=1$ (odd case), then there is only the singleton path (*x*, *y*); if $d=2$ (even case), then we can either form the path $(x,y)\rightarrow (y,x)$ of end-points (*x*, *x*) or the path $(y,x)\rightarrow (x,y)$ of end-points (*y*, *y*). So the base case is verified. Now we assume the Lemma holds true for a generic $d > 2$ and we want to prove it for $d+1$. If *d* is even, then $d+1$ is odd and we can either have a path $(x,x)\rightarrow (x,y)$ or a path $(x,y)\rightarrow (y,y)$. In both cases the end-points are (*x*, *y*). Symmetrically: if *d* is odd, then $d+1$ is even and we can either have a path $(x,y)\rightarrow (y,x)$ with end-points (*x*, *x*) or a path $(y,x)\rightarrow (x,y)$ with end-points (*y*, *y*). $\square$

Using Lemma 1 we can simplify *G* using a routine preprocess as follows.

- All nodes from the sets $E_{x,x}$ are oriented to form a single path of end-points (*x*, *x*). Hence all these nodes are removed from *G* and replaced with a new node (*x*, *x*). This new node is then merged to another node (*x*, *y*), if any, into a two-node path of end-points (*x*, *y*).
- All nodes from sets $E_{x,y}$ are oriented to form a single path of end-points (*x*, *y*) if $|E_{x,y}|$ is odd or two paths, both of end-points (*x*, *y*) if $|E_{x,y}|$ is even. In this latter case, two equal nodes (*x*, *y*) are added to *G*.

After preprocess, *G* has the following form: there are no nodes of the form (*x*, *x*) and nodes of the form (*x*, *y*) appear at most twice. Without loss of generality, we are going to assume that *G* has this form from now on.

After preprocess, two sets are built (lines 3–6 of Algorithm 3), *I* and *U*, that are manipulated by the subsequent steps merge-even and greedy-cover. The set *I* is the collection of all incidence sets $I_w$, $\forall \, w \in \mathcal {W}$. Note that, since nodes (*w*, *w*) are not present in *G* after preprocess, $n(I_w)=|I_w|$ for any *w*. The set *U* represents the set of all nodes that are still to be added by the algorithm to some path of $C^*$.

Whenever a node $u = (x,y)$ is to be inserted in *G*, *u* is actually added to *U*, $I_x$, and $I_y$. This steps are illustrated in $\textsc {Insert} ({u,I,U})$ (Algorithm 5). Symmetrically, we use $\textsc {Erase} ({u,I,U})$ to erase the node $u = (x,y)$ from *U*, $I_x$, and $I_y$.

**Fig. 5:**
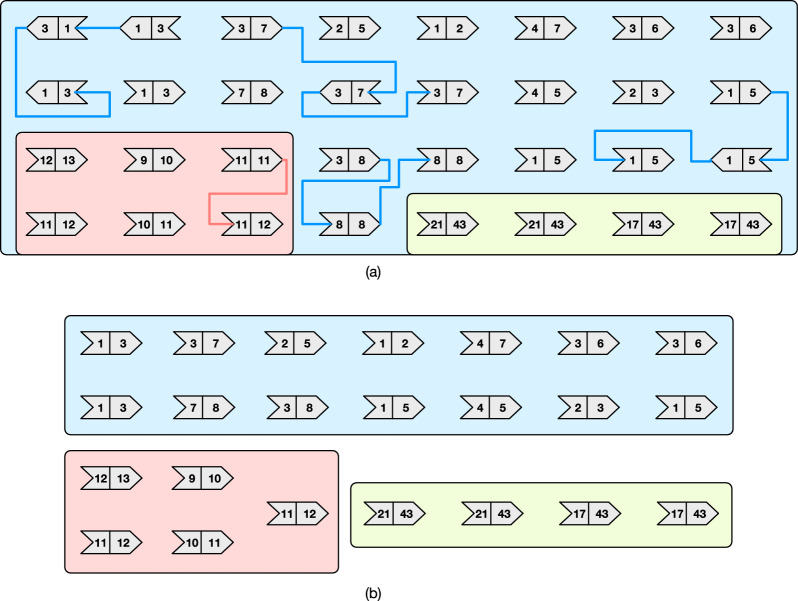
Graph simplification steps **a** for the graph from Fig. 4 and the resulting simplified graph **b**

#### *Example 2*

Figure 5 shows the simplification steps for the graph *G* from Fig. 4. Let $G'$ be the simplified graph. In the example graph *G* we have: $|E_{1,3}|=4$, hence $G'$ has two nodes of end-point (1, 3); also $|E_{1,5}|=4$, hence $G'$ has two nodes of end-point (1, 5); $|E_{3,7}|=3$, hence $G'$ has a single node (3, 7); $|E_{11,12}|=|E_{21,43}|=|E_{17,43}|=|E_{3,6}|=2$, hence $G'$ has all the nodes in these sets.

### Odd-frequency end-points

Let us first consider the odd-frequency end-points, i.e., those end-points *w* such that $|I_w|$ is odd.

#### **Lemma 2**

*If* $|I_w|$ *is odd then* *w* *appears as end-point of some path in* $C^*$.

#### *Proof*

Since each node has two matching sides, one node *u* in $I_w$ will remain unmatched. Hence *u* will be an end-point of some path in $C^*$. $\square$

Let $\mathcal {W}$ be defined after preprocess of *G*. We partition $\mathcal {W}$ into two sets, $\mathcal {W}_{odd}$ and $\mathcal {W}_{even}$: if $|I_w|$ is odd, then $w \in \mathcal {W}_{odd}$; otherwise, $w \in \mathcal {W}_{even}$.

#### **Lemma 3**

$$|\mathcal {W}_{odd}|$$ *is even.*

#### *Proof*

Observe that $\sum _{w \in \mathcal {W}} |I_w|$ is even and equal to 2|*G*| because each node has two end-points. Since $\mathcal {W} = \mathcal {W}_{odd} \cup \mathcal {W}_{even}$ and $\mathcal {W}_{odd} \cap \mathcal {W}_{even} = \varnothing$, the above sum can be re-written as $\sum _{w \in \mathcal {W}} |I_w| = \sum _{w \in \mathcal {W}_{odd}} |I_w| + \sum _{w \in \mathcal {W}_{even}} |I_w| = 2|G|.$ It follows that also $\sum _{w \in \mathcal {W}_{odd}} |I_w| = 2|G| - \sum _{w \in \mathcal {W}_{even}} |I_w|$ must be even since it is obtained by difference of even quantities. Since each term in the sum $\sum _{w \in \mathcal {W}_{odd}} |I_w|$ is odd by definition, the whole sum is even if and only if $|\mathcal {W}_{odd}|$ is even, as the sum of an odd number of odd numbers is odd. $\square$

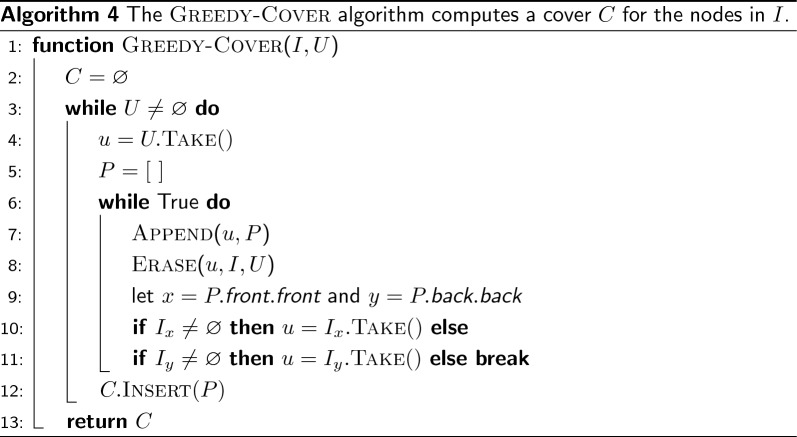



#### **Lemma 4**

*If* $\mathcal {W}_{even} = \varnothing$ *then* $|C^*| = |\mathcal {W}_{odd}|/2$ *and the* greedy-cover *algorithm (Algorithm 4) is optimal.*

#### *Proof*

For Lemma 2, any $w \in \mathcal {W}_{odd}$ will appear as end-point of some path in $C^*$. Since each path must necessarily have two end-points, then $|C^*| = |\mathcal {W}_{odd}|/2$.

**Fig. 6:**
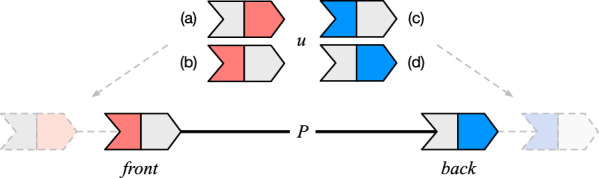
A graphical visualization of line 7 in Algorithm 4 which extends the path *P* with a node *u*. When *P* is not empty, four different cases can arise, as illustrated in **a**–**d**. In cases **b** and **d**, change-orientation(*u*) is called to match one of the two path’s end-points

Consider now the greedy-cover algorithm (Algorithm 4). We want to show it computes a solution *C* with exactly $|\mathcal {W}_{odd}|/2$ paths. At the beginning of each iteration of the main while-loop (lines 3–12), the algorithm takes an unvisited node from *U* (line 4) and begins a new path *P* from there. Nodes are appended to either the front or the back of *P*, for as much as possible (inner while-loop in the lines 6–11). For each node $u=(x,y)$ appended to *P* (see Fig. 6), it is also removed from *U*, $I_x$, and $I_y$ with $\textsc {Erase} ({u,I,U})$. Suppose now *P* cannot be extended any further. Then *P* must end with two weights that cannot appear as end-points of any other path since *P* is of maximal length. Hence, each time the inner while-loop (lines 6–11) ends, we have two weights less in $\mathcal {W}_{odd}$ that can appear as end-points. It follows that greedy-cover finds a solution with exactly $|\mathcal {W}_{odd}|/2$ paths. $\square$

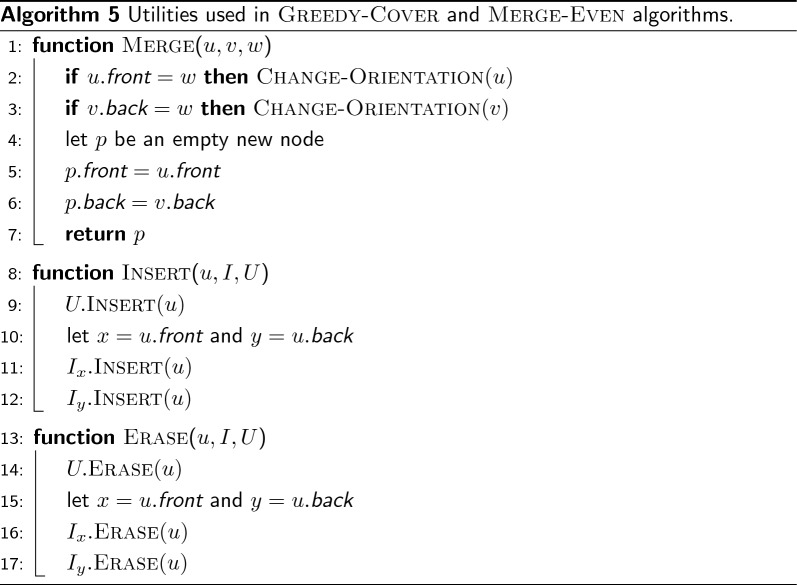



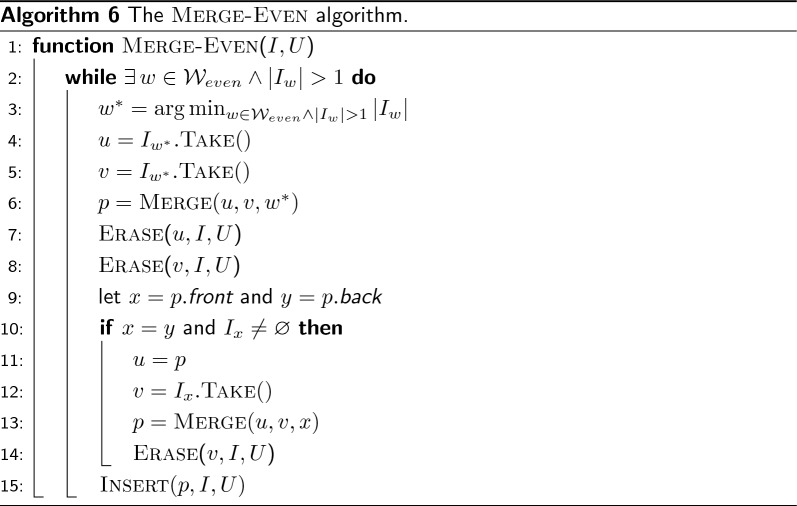



### Even-frequency end-points

Now note that the number of paths in $C^*$ is surely at least the number of connected components of *G* since there must be at least one path for each connected component. Let $\mathcal {C}$ be the set of connected components of *G*. The size of an optimal path cover for *G* is therefore $|C^*| = \sum _{c \in \mathcal {C}} |C^*(c)|$ where $C^*(c)$ is a minimum-size path cover for the connected component *c*.

#### **Lemma 5**

*Let* *c* *be a connected component of* *G*. *At the end of the* merge-even *algorithm (Algorithm 6),* *c* *is simplified to a graph where: either there is a single node, or* $I_w = \varnothing$ $\forall \, w \in \mathcal {W}_{even}(c)$.

#### *Proof*

We show that the merge-even algorithm maintains the following loop invariant: if $I_w \ne \varnothing$ and $w \in \mathcal {W}_{even}(c)$, there are at least two nodes *u* and *v* in $I_w$; otherwise $|c|=1$.

The invariant is true at the beginning of the algorithm before the first iteration, because: (1) either $\mathcal {W}_{even}(c) = \varnothing$, or (2) $|I_w| \ge 2$ for each $w \in \mathcal {W}_{even}(c)$ given that there are no nodes (*w*, *w*) in *c*, or (3) $|c|=1$.

Assume the invariant is true at the beginning of each iteration of the while-loop. We show it remains true after the iteration. At each iteration of the while-loop, let $w^*$ be a weight of minimum even frequency (line 3). Two nodes are removed from $I_{w^*}$ and merged into a parent node *p* with $\textsc {Merge} ({u,v,w^*})$. One of the following two cases can happen.

- The nodes *u* and *v* are such that $u=(x,w^*)$ and $v=(w^*,y)$, and *p* is therefore of end-points (*x*, *y*). Suppose either $|I_x|$ or $|I_y|$ is even (or both are). Without loss of generality, assume $|I_x|$ is even. Then it must be $|I_x| \ge |I_{w^*}| \ge 2$ because $w^*$ has minimum frequency, thus there are at least two nodes in $I_x$ if $|c|>1$ after merge, and the invariant is preserved (or *p* is the only node left in *c* and the invariant still holds). Otherwise, both $|I_x|$ and $|I_y|$ are odd. In this case, since both $|I_x|$ and $|I_y|$ remain odd after merge, nor *x* nor *y* will be considered by the algorithm at line 3.
- The nodes *u* and *v* are such that $u=(x,w^*)$ and $v=(w^*,x)$, and *p* is therefore of end-points (*x*, *x*). It could be that *p* is the only node where *x* appears, i.e., $I_x=\{(x,x)\}$, and the invariant would be violated unless $|c|=1$ after merge. However, the algorithm again merges *p* with any other node from $I_x$ (lines 10–14) if $I_x \ne \varnothing$ at line 10. Note that it must be that $I_x \ne \varnothing$ at line 10 if $|I_x|$ is odd, hence the second merge at line 13 is always possible. We now show that, if $I_x = \varnothing$ instead at line 10 then it must also be that $|c|=0$ and (*x*, *x*) will be the only node in *c* after the iteration, hence preserving the invariant. Assume by absurd that this is not the case, i.e., $I_x=\varnothing$ but $|c| \ne 0$ at line 10. Since $|c| \ne 0$, there must be other nodes in $I_{w^*}$ otherwise *c* cannot be a connected component at the beginning of the algorithm, i.e., $|I_{w^*}|>2$. But this would imply that $|I_x|<|I_{w^*}|$ because $|I_x|=2$ before merge, which contradicts the hypothesis that $w^*$ is a minimum even frequency weight. The algorithm therefore maintains the invariant that there are no nodes (*x*, *x*) in $I_x$, thus $|I_x| \ge 2$ unless $|c|=1$.

In all cases, it is easy to see that at each iteration: the parity of $|I_x|$ and $|I_y|$ do not change, $|I_{w^*}|$ decreases by 2, and the number of nodes in *c* decreases by 1 or 2. Summing up, merging on a weight of minimum even frequency preserves the loop invariant.

At the end of the algorithm, since at every iteration the even frequency of a weight is decreased by 2, either $I_w = \varnothing \, \forall \, w \in \mathcal {W}_{even}(c)$ or $|c|=1$ (or both). Note that if $\mathcal {W}_{odd}(c) = \varnothing$ at the beginning of the algorithm then the loop invariant guarantees that $|c|=1$ at the end of the algorithm. $\square$

**Fig. 7:**
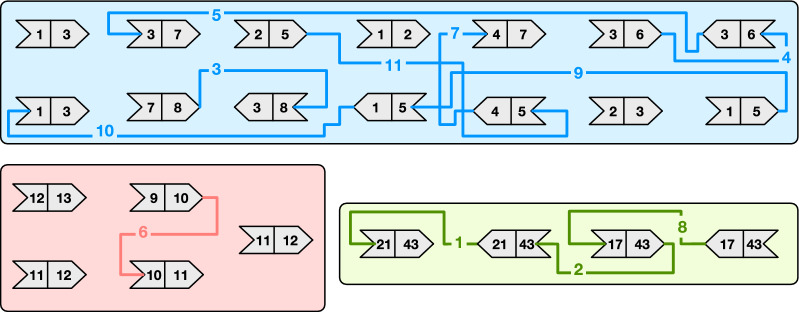
The merge operations performed by the algorithm merge-even on the graph from Fig. 5b. The order of the operations is represented by the numbers on the edges

**Fig. 8:**
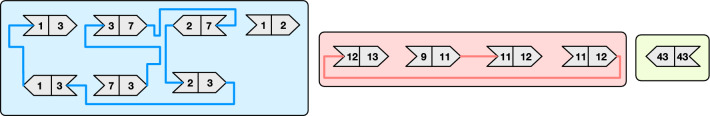
The execution of algorithm greedy-cover on the graph from Fig. 7 after the execution of merge-even. The resulting minimum-size path cover has $|C^*|=5$ paths

#### *Example 3*

Consider Fig. 7 showing the merge operations performed by algorithm merge-even. In the input graph, there are 8 weights whose frequency is even: 4, 5, 6, 8, 10, 17, 21, 43. After the execution of merge-even, each of the three connected components of the graph has no node whose weights have even frequency, except for the one with the green background whose nodes all have weights with even frequency, according to Lemma 5. Indeed, note that the green component is simplified to a single node. Lastly, Fig. 8 shows the execution of the algorithm greedy-cover after the action of merge-even. In this case, the final (optimal) path cover has 5 paths of end-points: (3, 7), (1, 2), (9, 12), (11, 13), and (43, 43).

### The final algorithm

We can now state and prove the optimality of the min-cover algorithm.

#### **Theorem 1**

*Let* $\mathcal {C}_{even}$ *be the set of connected components of* *G* *whose nodes only have end-points of even frequency. Then* $|C^*| = |\mathcal {C}_{even}| + |\mathcal {W}_{odd}|/2$ *and the* min-cover *algorithm (Algorithm 3) is optimal*.

#### *Proof*

Consider the min-cover algorithm (Algorithm 3). Since any pair of connected components have disjoint set of nodes by definition, $|C^*| = \sum _{c \in \mathcal {C}} |C^*(c)| = \sum _{c \in \mathcal {C}_{even}} |C^*(c)| + \sum _{c \notin \mathcal {C}_{even}} |C^*(c)|$. If $c \in \mathcal {C}_{even}$ then $\mathcal {W}_{odd}(c)=\varnothing$ and $C^*(c)=1$ for Lemma 5 since *c* will be simplified to one single node by merge-even. Hence, $\sum _{c \in \mathcal {C}_{even}} |C^*(c)| = |\mathcal {C}_{even}|$. If $c \notin \mathcal {C}_{even}$ then after merge-even there will only be nodes with odd weights in *c*. Thus, for Lemma 4, $C^*(c)=|\mathcal {W}_{odd}(c)|/2$ and $\sum _{c \notin \mathcal {C}_{even}} |C^*(c)| = |\mathcal {W}_{odd}|/2$. In conclusion, the min-cover algorithm is optimal. $\square$

### Time and space complexity

If we use hash tables to implement the sets *I* and *U*, then the operations take, insert, and erase, are all supported in *O*(1) on average. Also append can be performed in constant amortized time using a double-ended queue to represent the path *P*. The merge function in Algorithm 5 obviously takes constant time.

With these remarks in mind, it follows that the min-cover algorithm runs in $\Theta (m)$ amortized time, where $m=|G|$ is the number of nodes in the input graph *G*.

In fact, the preprocess routine can be implemented by sorting the *m* nodes in *O*(*m*) time with radix sort, and scanning the nodes in sorted order. The greedy-cover algorithm also takes *O*(*m*) time because each node is visited and appended to a path exactly once. The complexity of the merge-even algorithm (Algorithm 6) critically depends, instead, on the complexity of the step at line 3—the identification of the weight of minimum even frequency. In “Reporting the minimum weight in constant time” section we will show how to perform this operation in *O*(1) time. Hence, since the number of nodes in *G* reduces by 1 or 2 at each iteration of the while-loop and all operations in the body of the loop take constant time, the complexity of merge-even is *O*(*m*).

Lastly, the algorithm also consumes $\Theta (m)$ space because: (1) at most 2*m* nodes (and at least *m*) are inserted in *I* and exactly *m* in *U* during the initialization (lines 5–6 of min-cover); (2) the merge-even algorithm creates at most 2*m* new nodes.
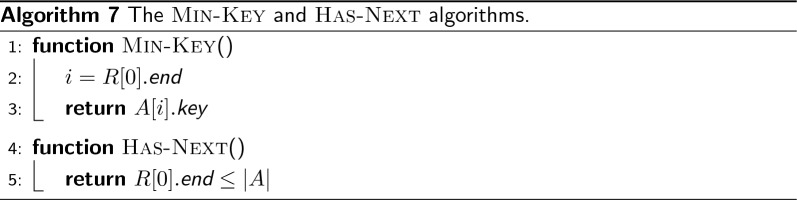

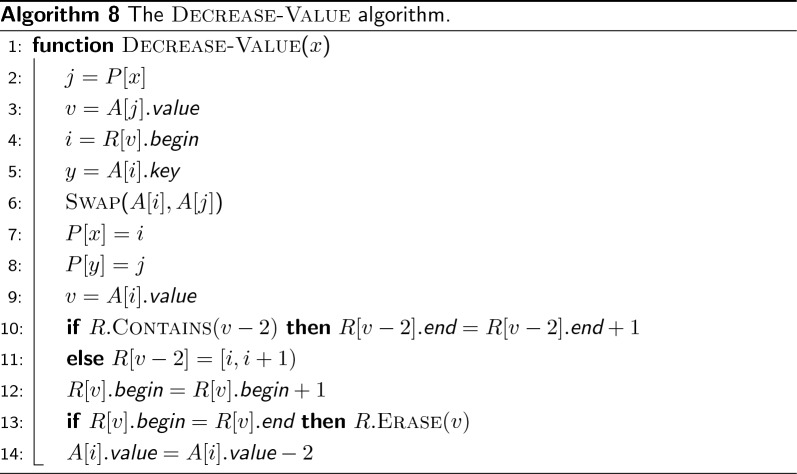


**Fig. 9:**
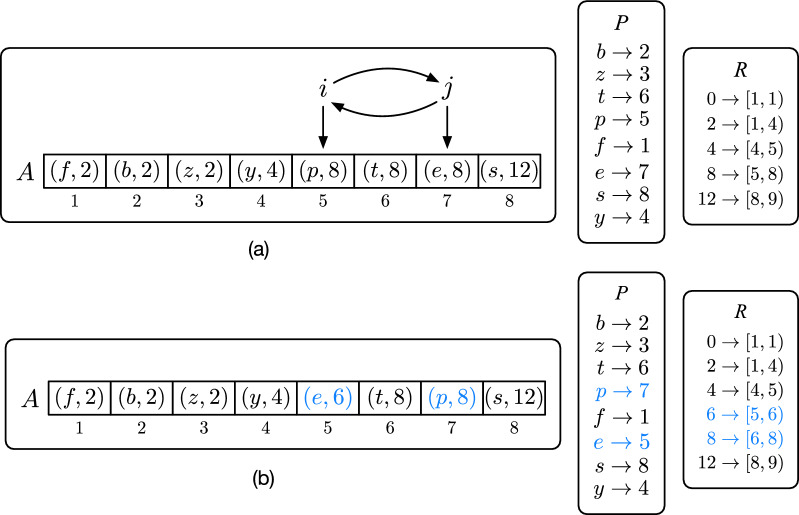
In **a**, an example array *A* with 8 pairs. In **b**, we show how *A*, *P*, and *R* are updated by decrease-value(*e*). After $$\textsc {Swap} ({i,j})$$ and the decrease of the value of *e* from 8 to 6, there is no element 6 in *R*, hence $$6 \rightarrow [5,6)$$ is added to *R* (line 11 of Algorithm 8) and $$R[8].begin $$ incremented by 1. Note that if the value of *y* were 6, then the test at line 10 would have succeeded, hence $$R[6].end $$ would have been updated

## Reporting the minimum weight in constant time

In this section we solve the following problem. We have an array *A* of $$(key ,value )$$ pairs, initially sorted by $$value $$. Keys are all distinct and values are even integers larger than or equal to 2. We want to answer min-key queries over *A*, i.e., to report the key of minimum value, under the condition that the value of a key *x* can be decreased by 2 with a decrease-value(*x*) operation.

Since we use a solution to this problem to implement the reporting of the weight of minimum even frequency in the merge-even algorithm (line 3 of Algorithm 6), we work under the assumption that decrease-value(*x*) cannot be called if the value associated to the key *x* is already 0. The minimum values in *A* can therefore be 0 but we want to report the key with minimum value larger than 0.

Let *K* and *V* be the set of keys and values of *A* respectively. We use two linear-space maps (implemented as hash tables), $$R: V \rightarrow \{1,\ldots ,|A|\}^2$$ and $$P: K \rightarrow \{1,\ldots ,|A|\}$$. Before answering any query on *A*, *R* and *P* are initialized such that $$R[v] = [begin ,end )$$ indicates that the values of the elements $$A[begin ,end )$$ are all equal to *v*, and such that $$P[x]=i$$ indicates that $$A[i].key =x$$. Also we add the special value 0 to *R* and let $$R[0] = [1,1)$$ at the beginning.

We want to show that the algorithm decrease-value (Algorithm 8) maintains the following invariants: (1) *A* is sorted by *value*; (2) all the values of the elements $$A[begin ,end )$$ are equal to *v* if $$R[v] = [begin ,end )$$ for each $$v \in V \cup \{0\}$$; (3) if $$P[x]=i$$ then $$A[i].key =x$$ for each key $$x \in K$$. If these invariants hold true after each call of decrease-value, then $$R[0].end $$ is the position of the key of minimum value larger than 0 and min-key can simply return $$A[R[0].end ].key $$ in *O*(1).

The invariants are all trivially satisfied by construction before answering any query: *A* is sorted and $$R[0].end =1$$, hence $$A[1].key $$ is the key of minimum value.

Suppose now that the invariants are true for some $$1 < R[0].end \le |A|$$. We want to show that they are also true after decrease-value(*x*). The position *j* of *x* is determined at line 2 and its value *v* at line 3. By assumption, $$v \ge 2$$. Then the algorithm identifies the beginning of the range of values equal to *v* in *A*, i.e., position *i*. It then swaps *A*[*i*] with *A*[*j*] and update *P* consequently so that, at line 9, *i* indicates the position of the key *x* whose value *v* has to be decreased by 2. Hence, lines 2-9 maintain the invariant that *A* is sorted by *value* as well as the invariants on *R* and *P*. Before decreasing the value $$A[i].value $$ by 2 at line 14, the algorithm adjusts *R*[*v*] and $$R[v-2]$$. Since the value *v* is going to be decreased by 2, $$R[v-2].end $$ has to be increased by 1 and, symmetrically, $$R[v].begin $$ has to be decreased by 1. Whenever we decrease a value, it is possible that $$v-2$$ does not yet exist in *R*. If that is the case, then $$R[v-2]$$ is initialized with $$[i,i+1)$$ at line 11, which is correct because *i* is the position of the key *x* whose value has to be decreased. Whenever we increase the beginning of a range instead, it is possible that the range is exhausted, i.e., $$R[v].begin = R[v].end $$. If that is the case, it means that there is only one key with value *v* and, since *v* is going to be decrease, *v* is correctly erased from *R*. Figure 9 shows an example for an array *A* of 8 pairs and how the invariants on *A*, *P*, and *R* are preserved after decrease-value(*e*).

In conclusion, all the invariants are preserved after every call of decrease-value. Therefore—if all values are decreased to 0—then there will only be the value 0 in *R* and $$R[0] = [1,|A|+1)$$. Note that each step of decrease-value takes *O*(1) time since *R* and *P* are implemented with hash tables, hence the overall time of decrease-value is *O*(1).
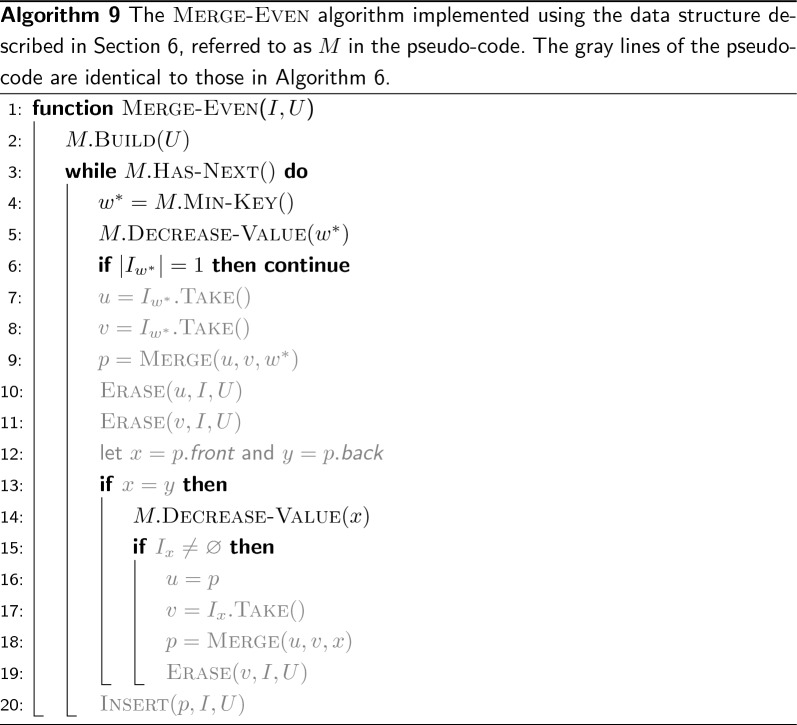


Let *M* be the data structure holding the array *A*, the maps *R* and *P*, and exposing the functions build, has-next, min-key, and decrease-value. Algorithm 9 illustrates how to use the data structure to implement the merge-even algorithm previously described as Algorithm 6. The data structure is built from the nodes in *U* by letting the weights be the keys in *A* and the frequencies of the weights be the values in *A*. Since there are at most $m=|G|$ nodes in *U*, the initialization of *A*, *R*, and *P* takes *O*(*m*) time and the whole data structure consumes *O*(*m*) space.

*Note* A solution to the problem of maintaining a sorted list of integers subject to increments/decrements was also given by Knuth [42] to implement the *adaptive* Huffman coding algorithm, but using a different combination of elementary data structures. The presentation given here is specifically suited for Algorithm 6 (see also Algorithm 9).

## Experiments

In this section we evaluate the proposed weight compression scheme for SSHash and compare it to several competitive baselines. We first describe our experimental setup. Experiments were run using a server machine equipped with a Intel i9-9900K CPU (clocked at 3.60 GHz) and 64 GB of RAM. All the tested software was compiled with gcc 11.2.0 under Ubuntu 19.10 (Linux kernel 5.3.0, 64 bits), using the flags -O3 and -march=native. Our implementation of SSHash is written in C++17 and available at https://github.com/jermp/sshash.

All timings were collected using a single core of the processor. The dictionaries are loaded in internal memory before executing queries. For all the experiments, we fix *k* to 31.

### Datasets

 We use the following genomic collections: E-Coli and C-Elegans are, respectively, the full genomes of *E. Coli* (Sakai strain) and *C. Elegans* that were also used in the experimentation by Shibuya et al. [27]; S-Enterica-100 is a pan-genome of 100 genomes of *S. Enterica*, collected by Rossi et al. [43]; Human-Chr-13 is the 13-th human chromosome from the genome assembly GRCh38. Table 1 reports some basic statistics for the collections. The weights were collected using the tool BCALM (v2) [44] without any filtering (option -all-abundance-counts). In general, note the very low empirical entropy of the weights, $H_0(W)$. This is expected since most $k$-mers actually appear once for large-enough values of *k*. Instead, the weights on the pan-genome S-Enterica-100 have much higher entropy due to the fact that many $k$-mers have weight equal to the number of genomes in the collection (in this specific case, equal to 100). This is useful to test the effectiveness of our encoding on both low- and high- entropy inputs.

All datasets, in raw and pre-processed form, are available on Zenodo at https://zenodo.org/record/7772316.

**Table 1:** Some basic statistics for the datasets used in the experiments, for $k=31$, such as: number of distinct $k$-mers (*n*), number of distinct weights ($|\mathcal {D}|$), largest weight (*max*), expected weight value (*E*), and empirical entropy of the weights ($H_0(W)$)

Dataset	*n*	${|\mathcal {D}|}$	${\lceil \log _{2}|\mathcal {D}|\rceil }$	${max }$	${\lceil \log _{2}max \rceil }$	*E*	${H_{0}(W)}$
E-Coli	5,235,781	22	5	27	5	1.05	0.206
S-Enterica-100	12,408,741	620	10	7956	13	38.94	4.155
Human-Chr-13	90,911,778	806	10	6354	13	1.08	0.160
C-Elegans	94,006,897	398	9	3478	12	1.07	0.223

**Table 2:** Space for the weights in SSHash reported in bits/$k$-mer, *before* and *after* the run-reduction optimization

Dataset	$H_0(W)$	Before		After	
E-Coli	0.206	0.017	($12.35\times$)	0.014	($15.10\times$)
S-Enterica-100	4.155	0.464	($8.96\times$)	0.328	($12.66\times$)
Human-Chr-13	0.160	0.135	($1.18\times$)	0.108	($1.50\times$)
C-Elegans	0.223	0.069	($3.23\times$)	0.055	($4.05\times$)

For reference, we also report how many times the achieved space is better than the empirical entropy of the weights $H_0(W)$

**Table 3:** The number of input strings (*m*) for SSHash as computed by UST [30], the number of runs (*r*) computed by the min-cover algorithm (Alg. 3)

Dataset	*m*	*r*	Alg.3 (ms)	Alg. 3 (ns/node)
E-Coli	2115	3720	0.2	54
S-Enterica-100	111,254	208,700	9.2	82
Human-Chr-13	266,263	461,917	14.2	54
C-Elegans	140,422	247,941	7.3	52

The last two columns show the run-time of min-cover, in total milliseconds (ms) and average nanoseconds per node (ns/node)

### Weight compression in SSHash

 We now consider the space effectiveness of the encoding scheme described in “Representing runs of weights” section. Table 2 reports the space as average bits per $k$-mer: we see that, in all cases, the space is well below the empirical entropy lower bound $H_0(W)$—usually below by several times. The optimization strategy described in “The problem of reducing the number of runs” section brings further advantage. (The space shown is comprehensive of the $|\mathcal {D}|\lceil \log _2max \rceil$ bits used to represent the distinct weights in the collection. Note that this space takes a negligible fraction of the total space since $|\mathcal {D}|$ is very small as reported in Table 1).

Table 3, instead, shows the performance of the path cover Algorithm 3. As already mentioned in “Representing runs of weights” section, the set of strings indexed by SSHash is obtained by building a spectrum-preserving string set (SPSS) from the raw genome, using the algorithm UST [30] over the output of BCALM [44]. (At the code repository https://github.com/jermp/sshash we provide further details on how to take these preliminary steps before indexing with SSHash). The number of strings in each collection, *m*, determines the run-time of Algorithm 3 whose complexity is $\Theta (m)$. The linear-time complexity is evident from the reported timings and makes the algorithm very fast, taking $\approx$50-80 nanoseconds per node.

In Appendix we report additional experimental results.

### Overall comparison

 In Table 4 we show a comparison between the following weighted $k$-mer dictionaries:

- The dBG-FM index [45]^2^ based on the popular FM-index [22]. In particular, this representation implements a weighted $k$-mer dictionary via the *count* query which returns the number of occurrences of a given $k$-mer in the input. The *count* query, in turn, is implemented using rank queries over the BWT. The dBG-FM implementation has a main trade-off parameter, *s*, to control the practical performance of rank queries. We test the values $s=32,64,128$.
- The cw-dBG [16]^3^ dictionary based on the data structure called BOSS [24]. Similarly to an FM-index, also cw-dBG has a trade-off parameter that we vary as $s=32,64,128$. (The authors used $s=64$ in their own experiments).
- The *non*-weighted SSHash itself coupled with the fast compressed static function (CSF) tailored for low-entropy distributions, proposed by Shibuya et al. [27].^4^ As reviewed in “Related work” section, a CSF does not represent the $k$-mers but just realizes a map from $k$-mers to their weights. Such map is collision-free only over the set of $k$-mers that was used to actually build the function. Therefore, we use SSHash as an efficient dictionary for the $k$-mers and the CSF to represent the weights. The authors proposed two different versions of their approach, BCSF and AMB, with different space/time trade-offs.
- The weighted SSHash dictionary proposed in this work, which we refer to as w-SSHash in the following, *after* the run-reduction optimization (Tables 2 and 3). We use the *regular* index variant of SSHash. The main parameter of the index—the *minimizer* length—is always set to $\lceil \log _4 N \rceil + 1$ where *N* is the number of nucleotides in the SPSSs of the datasets, following the recommendation given in the previous paper [18]. Therefore, we use the following minimizer lengths: 13, 14, 15, and 15, for respectively, E-Coli, S-Enterica-100, Human-Chr-13, and C-Elegans. Also the AMB algorithm by Shibuya et al. [27] is based on minimizers and we use the same lengths.

**Table 4:** Dictionary space in average bits/$k$-mer (bpk) and total MB, and query time in average μs/$k$-mer (qtm)

Dictionary	E-Coli			S-Enterica-100			Human-Chr-13			C-Elegans		
	bpk	MB	qtm	bpk	MB	qtm	bpk	MB	qtm	bpk	MB	qtm
dBG-FM, $s=128$	3.20	2.00	12.42	118.23	174.90	14.00	3.23	34.97	14.94	3.18	35.60	15.44
dBG-FM, $s=64$	4.02	2.51	6.57	147.84	218.70	9.43	4.07	44.07	9.98	4.01	44.89	9.60
dBG-FM, $s=32$	5.65	3.53	3.74	206.53	305.51	7.03	5.73	62.15	7.25	5.67	63.49	7.10
cw-dBG, $s=128$	2.79	1.82	96.84	5.43	8.42	111.43	2.80	31.77	92.80	2.77	32.54	119.73
cw-dBG, $s=64$	2.86	1.87	62.97	5.58	8.66	76.74	2.86	32.55	67.63	2.84	33.34	77.72
cw-dBG, $s=32$	2.99	1.96	46.49	5.87	9.11	62.21	2.99	34.02	54.48	2.97	34.87	56.67
SSHash+BCSF	5.02	3.29	0.48	11.43	17.73	0.52	6.12	69.55	0.88	5.94	69.80	0.9
SSHash+AMB	4.85	3.17	0.57	9.15	14.19	0.68	6.05	68.75	1.06	5.82	68.39	1.07
w-SSHash	4.80	3.14	0.35	5.97	9.26	0.46	6.04	68.66	0.82	5.75	67.52	0.85
**SSHash**	**4.79**	**3.14**	**0.32**	**5.63**	**8.73**	**0.39**	**5.93**	**67.39**	**0.73**	**5.69**	**66.86**	**0.77**

For reference, we report in bold the space and time of SSHash *without* the weight information

We did not compare against deBGR [17] and Squeakr [13] as the authors of cw-dBG showed in their experimentation [16] that both tools take considerably more space than cw-dBG, e.g., one order of magnitude more space. Here, we are interested in a good balance between space effectiveness and query efficiency. The same consideration applies to the popular $k$-mer counting tool KMC3 [12] which stores both $k$-mers and their abundances in a hash table without compression.

To measure query-time—the time it takes to retrieve the weight *w*(*g*) given the $k$-mer *g*—we sampled $10^6$ $k$-mers uniformly at random from the collections and use them as queries. We report the mean between 5 measurements. Half of the queries were transformed into their reverse complements to make sure we benchmark the dictionaries in the most general case.

The space of w-SSHash is generally competitive with that of the fastest variant of dBG-FM ($s=32$), but w-SSHash has (more than) one order of magnitude better query time. Note that on S-Enterica-100 the dBG-FM index is space-inefficient since it redundantly represents many repeated $k$-mers. Using a higher sampling rate reduces the space of dBG-FM at the price of slowing down query-time; however, the most space-efficient variant tested ($s=128$) is not even $2\times$ smaller than w-SSHash.

The cw-dBG index is the smallest tested dictionary. Its space effectiveness is comparable to that of dBG-FM $s=128$, and indeed generally twice as better as that of w-SSHash. The price to pay for this enhanced compression ratio is a significant penalty at query-time. Indeed, w-SSHash can be two order of magnitude faster than cw-dBG. Consider, for example, the two dictionaries built for S-Enterica-100: we have 0.5 vs. $\approx$60–110 μs per query.

The two CSFs, BCSF and AMB, make SSHash consistently slower and larger than w-SSHash This comparison motivates the need for a unified data structure to handle efficiently both the $k$-mers *and* the weights, like w-SSHash. While the increase in space due to the CSF is not much for the low-entropy datasets because both BCSF and AMB are very space-efficient in those cases, the gap is more evident on S-Enterica-100.

As a last note, observe that there is no significant slowdown in accessing the weights in w-SSHash compared to a simpler membership query (the time reported in shaded color in Table 4), hence proving the RLE-based scheme to be efficient too and not only very effective.

## Conclusions

In this work we extended the recent SSHash [18] dictionary to also store the weights of the $k$-mers in compressed format. In particular, we represented the weights using compressed *runs* of equal symbols. While using run-length encoding to compress highly repetitive sequences is not novel per se and indeed a folklore strategy at the basis of many other data structures, this allows to use a very small extra space (e.g., much less than the empirical entropy of the weights) on top of SSHash with only a slight penalty at retrieval time. The crucial point is that it is possible to use run-length encoding because SSHash *preserves the (relative) order* of the $k$-mers in the indexed sequences. The main practical take-away is, therefore, that SSHash handles weighted $k$-mer sets in an *exact* manner without noticeable extra costs. Our software is publicly available to encourage its use and reproducibility of results.

We also introduced the concept of *end-point weight graph* and showed its usefulness in reducing the number of runs in the weights. Precisely, we showed that minimizing the number of runs in a collection of sequences corresponds to the problem of computing a minimum-cardinality path cover for the end-point weight graph of the sequences. We presented an optimal algorithm that computes a cover of minimum size in linear-time (in the number of nodes of the graph). As a result of this optimization, the space spent to represent the weights is unlikely to be improved using run-length encoding.

Although several approaches in the literature [13, 26–28] also consider *approximate* weights, we did not pursue this direction here as the weights are already encoded space-efficiently in SSHash and in an *exact* way, so there may be no need for approximation.

The distribution of weights in large collections is usually expected to be very skew, i.e., most $k$-mers actually appear once and few of them repeat many times [26, 27]. A common strategy to save space is then to avoid the representation of the most frequent weight(s). Note that, since we represent runs of weights and not the individual weights, we are already optimizing (potentially very large) sub-sets of weights equal to the most frequent one. That is, run-length encoding is also a good match for such skew distributions.

## Data Availability

The SSHash software is available on GitHub at https://github.com/jermp/sshash. The datasets used in this article are available on Zenodo at https://zenodo.org/record/7772316.

## Appendix: Additional experimental results

**Table 5:** The performance of the min-cover algorithm (Alg. 3) on the datasets Cod, Kestrel, Human, and Bacterial, for which we report the number of distinct $k$-mers (*n*) and the number of strings (*m*) after running UST [30] on the collections

Dataset	*n*	*m*	*r*	Alg. 3 (sec)	Alg. 3 (ns/node)
Cod	502,465,200	2,406,681	4,183,202	0.14	57
Kestrel	1,150,399,205	682,444	1,140,743	0.03	49
Human	2,505,445,761	13,013,742	22,680,047	0.76	59
Bacterial	5,350,807,438	26,448,286	56,662,230	1.64	62

The performance of the algorithm is expressed as the number of runs (*r*) after the run-reduction optimization and running time (in total seconds and average ns/node)

**Table 6:** The performance of w-SSHash on the permuted string collections Cod, Kestrel, Human, and Bacterial

Dataset	$H_0(W)$	bpk		GB	qtm
Cod	0.441	6.98 + 0.19	($2.35\times$)	0.45	1.3
Kestrel	0.089	6.49 + 0.02	($3.80\times$)	0.94	1.1
Human	0.453	8.28 + 0.22	($2.06\times$)	2.66	1.7
Bacterial	1.890	8.22 + 0.24	($7.81\times$)	5.66	1.7

We report the empirical entropy of the weights ($H_0(W)$), the dictionary space in average bits/$k$-mer (bpk) and total GB, and query-time in average μs/$k$-mer (qtm). The space is indicated as $x+y$, where *x* is the space of SSHash (without the weights) and *y* is the space for the encoding of the weights. In parentheses we report the space reduction of the encoded weights compared to the empirical entropy of the weights

In Tables 5 and 6 we report the performance of the min-cover algorithm and of w-SSHash on four additional, larger, collections that we also used in our previous work [18], namely the full genomes of *G. morhua* (Cod), *F. tinnunculus* (Kestrel), and *H. sapiens* (Human), and a collection of more than 8000 bacterial genomes (Bacterial) [46]. Precisely, the results in Table 6 are for regular w-SSHash dictionaries with minimizer lengths equal to 17, 17, 20, and 20, for respectively, Cod, Kestrel, Human, and Bacterial.

## Footnotes

1 Elias-Fano represents a monotone integer sequence *S*[1..*n*] with $S[n] \le U$ in at most $n\lceil \log _2(U/n)\rceil +2n$ bits. With *o*(*n*) extra bits it is possible to decode any *S*[*i*] in constant time and support predecessor queries in $O(\log (U/n))$ time. For a complete description of the method, we point the reader to the survey by Pibiri and Venturini [33, Section 3.4]. We also remark that Elias-Fano has been recently used in many compressed, practical, data structures, e.g., inverted indexes [33–37], tries [38–40], and full-text indexes [41].

2 https://github.com/jts/dbgfm.

3 https://github.com/nicolaprezza/cw-dBg.

4 https://github.com/yhhshb/locom.
